# Association Between Serum Adiponectin and HDL-C in Type II Diabetic Patients

**DOI:** 10.5539/gjhs.v7n2p243

**Published:** 2014-11-16

**Authors:** Nasser Shokri Kalehsar, Tagi Golmohammadi

**Affiliations:** 1 Department of Biochemistry, Ardabil branch, Islamic Azad University, Ardabil, Iran; 2 Department of Medical Biochemistry, Faculty of Medicine, Tehran University of Medical Sciences, Iran

**Keywords:** adiponectin, high-density lipoprotein (HDL-C), type II diabetes

## Abstract

**Introduction:**

Adiponectin has an important role in the metabolism of lipids and carbohydrates, and it also has a role in vascular biology. The aim of this study was to determine the association of adiponectin with HDL-C in type II diabetic patients and healthy individuals.

**Methodology:**

This was a case control study. After 12 hours of fasting, the patients’ blood samples was taken, and the serum was separated in a centrifuge tube. The concentration of adiponectin in the serum was measured using ELISA kits. The same procedure also was performed for the members of the control group.

**Findings:**

The adiponectin levels were signifcantly different between the two groups, i.e., the type II diabetic patients and the individuals in the control group who were healthy nondiabetics (P<0.001) and (t=5.93). There was a significant difference in the HDL-C values between the two groups (P<0.001) and (t=11.30). A significant relationship also was found between the amounts of adiponectin and HDL-C (P≤0.001).

**Conclusions:**

The results of this study can be used to identify individuals at risk for type II diabetes, and to control the risk factors for type II diabetes and cardiovascular diseases by increasing the levels of adiponectin in the blood.

## 1. Introduction

One of the world’s major health problems is diabetes, particularly type II diabetes, which afflicts nearly 150 million people worldwide. A disproportionate number of those people, i.e., 84 million people with type II diabetes, live in developing countries. According to the World Health Organization’s (WHO’s) predictions, the cases of diabetes in these countries will increase by 170% from 1995 to 2025. This means that there will be approximately 227 million people with type II diabetes in developing countries in 2025 (Huang, 2003).

In developing countries, the number of people with diabetes is expected to increase by 150% in the next 25 years. This increase is due to the aging of world’s population, poor diets, excessive obesity, and unhealthy lifestyles. People who have diabetes in developing countries, usually are 35 to 64 years old (Hedayati Emami, 2001). Five percent of the total population of Iran, which is between 3 and 4 million people, have diabetes (Hedayati Emami, 2003). Although Iran, compared with other developing countries, ranks in the middle of the range for citizens with diabetes, the incidents of diabetes in Iran are increasing at a rate that is four times as great as the growth of the country’s population. Diabetes kills, nearly 1.5 million people per year, which amounts to 4,110 per day or three people per minute (Hedayati Emami, 2001). All these shocking statistics mean that diabetes is a very serious health issue worldwide. According to the Diabetes Prevention and Control Pilot Project that was conducted by the Management Center for Disease Control, the prevalence of diabetes II in the population is at least 3.6%, which means that in a population of 65 million people, more than 2.3 million people are estimated to be diabetic, and 90% of those people have type II diabetes (Asadi, 2003). According to the latest statistics published by the Ministry of Health in 2002, diabetes has killed 100,000 people in Iran. The costs associated with caring for people with diabetes in Iran were more than 700 billion Rials in 1996 (Hedayati Emami, 2001). It has been known for years that there is a strong relationship between obesity and diabetes and cardiovascular diseases, and it also is believed that adipose tissue is the major site of fat storage. Recent research has confirmed this belief and also has shown that adipose tissue functions as an endocrine organ, which has an important role in metabolic processes and inflammatory signals that control energy homeostasis (Yamauchi, 2003; Goldfine, 2003).

The aim of this study was to determine the association between the amount of serum adiponectin and HDL-C in type II diabetic patients.

## 2. Materials and Methods

This study was conducted in control-case format, and more than 100 cases of patients who were suspected of having type II diabetes were studied in Hazrat Imam Khomeini Hospital in Ardabil, Iran. Finally, after deleting the inappropriate and useless files, 50 people were used as the case subjects and 50 healthy people were used as controls. Both groups were investigated in terms of various factors, including age, gender, marital status, occupation, type of illness, duration of diabetes, family history of the disease, history of cardiovascular disease, duration of treatment, drugs being used, the use of anti-diabetic medications, anti-lipid drugs, BMI, FBS, CHO, TG, HDL, LDL, and adiponectin. Finally, the data were analyzed using SPSS statistical software. Table 1 shows the items used in the checklists of the patients and the control group.

**Table 1:** Checklist used to extract information from patients and controls

Row	Range	Row	Range
**1**	Disease type	11	Smoking
**2**	Other diseases	12	Sport
**3**	Time of diabetes diagnosis	13	(BMI
**4**	Duration of diabetes	14	((mg/dl FBS
**5**	Family history of diabetes	15	(mg/dl) CHO
**6**	Cardiovascular disease history	16	LDL-C (mg/dl)
**7**	Duration of treatment	17	HDL-C (mg/dl)
**8**	Prescribed Drugs	18	TG (mg/dl)
**9**	Use of anti-diabetic medication	19	adiponectin (µg/ml)

**10**	Use of anti-lipid drugs		

In this study, the patients included 50 people who were 30 years old or older who had type II diabetes. These patients had been referred to Imam Khomeini Hospital of Ardabil, where they were diagnosed with type II diabetes by endocrinologists. The members of the control group (n=50) were selected randomly among healthy adults who were older than 30. The patients were enrolled in the study after they understood and accepted the study conditions. The ages and genders of the control group were matched with those of the patients. Blood samples were taken from the patients and the mebers of the control group after 12 hours of fasting, and the serum was separated from the blood in a centrifuge tube. The serum samples were frozen at -18°C until the analyses were to be conducted. The concentrations of adiponectin in the serum samples from both groups were measured using ELISA kits. The amount of HDL-C and other lipid profiles (TG, LDL-C, and total cholesterol) and glucose were measured using the corresponding kits (Pars Azmoun Co. Tehran, Iran).

## 3. Results

Table 2 shows the comparison of the adiponectin levels in the patients and the controls. There was a significant difference in the adiponectin levels (P<0.001) between the two groups. There also was a significant difference between the HDL values of the two groups (P<0.001). In addition, there was a significant difference between the BMI values of the two groups BMI (P≤0.001).

**Table 2:** Compariosn of the results for the patients’ group and the control group

Row	Studied variables	Statistical Test	Result	P Value
1	Adiponectin	T student	Significant difference	**0.001**
2	HDL	T student	Significant difference	**0.001**
3	BMI	T student	Significant difference	**0.001**
4	FBS	T student	Significant difference	**0.001**
5	Cholesterol	T student	no significant difference	**0.71**
6	Triglycerides	T student	no significant difference	**0.38**
7	LDL	T student	Significant difference	**0.001**

The comparison of the FBS in the two groups showed that there was a significant difference between the two groups (P≤0.001). The results of comparison of the two groups also showed that there was no significant difference in their cholesterol levels (P<0.71). There was no significant difference in the triglycerides of the two groups (P<0.38). There was, however, a signiificant diffrence in the amount of LDL between the two groups (P<0.001).

The concentrations of serum adiponectin, HDL, LDL, CHO, BMI, FBS, total cholesterol, and triglycerides in both groups were compared using the T-test. The mean values of adiponectin and HDL in the group of healthy subjects were greater than those for the patients, and FBS and BMI in the patients were greater than they were in the healthy subjects.

## 4. Discussion

Higher levels of adiponectin and HDL in healthy individuals show that there is a correlation between these two variables, and when they are accompanied by high levels of FBS and BMI, patients are more susceptible to cardiovascular disease. Decreased values of HDL-C are associated with the formation of atheroma (fatty deposits on the walls of arteries), and, in a study conducted by Valsamkis, it was reported that serum adiponectin has a positive association with HDL-C, so this relationship may help prevent atherosclerosis and type II diabetes (Valsamakis, 2003).

Hotta et al. reported that, in type II diabetic patients, there is negative relationship between adiponectin and triglycerides and positive relationship between adiponectin and HDL-C (Hotta, 2000). Weyer et al. reported that hipoadiponectin in overweight individuals and in type II diabetic patients is closely associated with insulin resistance and hyperinsulinemia (Weyer, 2001).

Also, women tend to have higher levels of adiponectin than men. Adiponectin reduces the protein that transports fatty acids to the liver, which reduces the fatty acid content in the liver and the content of tigycerides in the liver. Consequently, the oxidation of fatty acids increases, and liver’s glucose output and synthesis of VLDL decrease (Chandran, 2003; Yamauchi, 2002).

The comparison of adiponectin levels in the patients’ group and the healthy group showed that there was no significant difference between them. Thus, one could conclude that even a family history of diabetes has no effect on the increase or decrease of this hormone.

Comparision of the adiponectin levels in type II diabetic patients and the control subjects showed that two groups had similar histories of cardiovascular disease, so one could conclude that a history of cardiovascular disease has no effect on the increase or decrease of this hormone. A comparison of the the mean levels of adiponectin in the patients’ group and the control group showed that the levels were similar even among those who were using anti-diabetes and anti-fat drugs, so one could conclude that such drugs have no effect on the levels of adiponectin.

Smoking and exercise effectively increase the levels of adiponectin. While these two variables were considered in this research, we did not calculate and interprete the impact of these variables on the secretion of adiponectin because the majority of subjects were non-smokers and did not participate in sports activities. Racial and ethnic characteristics also affect adiponectin levels, and the adiponectin levels are greater in some races, such as Caucasians. So, it seems apparent that lower levels of serum adiponectin increase the risk of diabetes and coronary heart disease in some communities and races (Chandran, 2003; Yamauchi, 2003).

## 5. Conclusions

The results showed that the mean value for serum adiponectin was greater than we expected it to be in healthy individuals. Increasing the levels of serum adiponectin may be used as a therapeutic strategy for the treatment of insulin resistance type II diabetes. In the future, adiponectin may be considered as an anti-obesity drug. A study of type clinical trials with the purpose of impact dyslipidemia is recommended as the enhancer drug HDL-C.
